# Neglected premotor neglect

**DOI:** 10.3389/fnhum.2014.00778

**Published:** 2014-10-15

**Authors:** Styrmir Saevarsson, Simone Eger, Maria Gutierrez-Herrera

**Affiliations:** ^1^Clinical Neuropsychology Research Group (EKN), Department of Neuropsychology, Bogenhausen Academical HospitalMunich, Germany; ^2^Department of Psychology, University of InnsbruckInnsbruck, Austria; ^3^Department Biology II Neurobiology, Graduate School of Systemic Neurosciences, University of Munich (LMU)Munich, Germany

**Keywords:** premotor neglect, directional action neglect, lesion-symptom mapping, neuroanatomy, assessment methods, neglect therapy

Unilateral neglect, or neglect for short, is commonly described as the failure to respond and attend to stimuli presented on the contralesional side. It cannot be explained by primary motor and sensory impairment (Heilman et al., 1987), and is usually caused by a stroke. Although neglect patients often recover spontaneously within several weeks, they demonstrate poorer amelioration and require longer hospitalizations following a stroke compared to stroke patients without the affliction (e.g., Buxbaum et al., 2004; Gillen et al., 2005). Many different subforms of neglect have been specified to date (e.g., Saevarsson et al., 2011). One of these, premotor neglect (PMN; also known as intentional motor neglect, directional action neglect, etc.; see Saevarsson, 2013a) denotes an intentional, voluntary, and directional (e.g. eye, hand, and head) motor bias from the ipsilesional side to an object in the contralesional side of space (Watson et al., 1978; Halligan and Marshall, 1989; Bisiach et al., 1990; Goodale et al., 1990; Heilman et al., 2008; Saevarsson, 2013a). For instance, patients may fail to reach an apple on their left side with their right hand (i.e., directional akinesia; Heilman et al., 1987) although they may be visually aware of the object. The foundation of PMN diagnosis is based on various studies that indicate performance improvement or decline when patients perform tasks that require directional movements under different visual conditions (see Saevarsson, 2013a for discussion). PMN is often seen alongside other neglect forms (in approximately 45% of cases), although exact incidence has not been specified (Saevarsson, 2013a). Unfortunately, many neglect reviews and empirical studies ignore PMN altogether (e.g., Saevarsson et al., 2008; Karnath, 2014), or report it merely as an unimportant accompaniment and not specific to neglect (e.g., Himmelbach and Karnath, 2003; Rossit et al., 2009a; Striemer and Danckert, 2013). For example, Himmelbach et al. (2007, p. 1980) claim that PMN is not a “consequence of spatial neglect but rather indicate[s] a phenomenon occurring in some of these patients as well as in other stroke patients (without neglect), i.e., a phenomenon occurring with (so far not further identified) brain damage.” In line with this view, the number of studies on PMN have decreased considerably since the 1990s (Saevarsson, 2013a). Conversely, many authors argue for the importance of PMN (e.g., Mattingley and Driver, 1997; Konczak and Karnath, 1998; Vossel et al., 2010; Saevarsson, 2013b) although non-neglect-based terms such as directional hypokinesia are often used. For instance, the most commonly applied neglect definition of Heilman et al. (1987) refers to PMN when describing the affliction. Controversially, current mainstream literature does not reject this description despite the fact that some authors seem to prefer “spatial” or “hemispatial” neglect as a synonym, although representational neglect is non-spatial in nature. The nature of PMN is poorly understood and may hold the key to advanced neglect assessment and rehabilitation (Punt and Riddoch, 2006; Saevarsson, 2013a), thus we argue for the existence and importance of PMN with regard to various clinical, neuroanatomical, and methodological issues.

Previous studies questioning the importance of PMN suffer from significant methodological limitations. This is partially due to difficulties in differentiating between similar PMN and visual neglect symptoms (see Saevarsson, 2013a for discussion). Performance on standard and PMN tests can be interpreted as indicating visual neglect (i.e., failure to notice items on the left side; e.g., Làdavas et al., 1993) and PMN (see Mattingley and Driver, 1997; Saevarsson, 2013a). Rossit et al. (2009a,b) revealed that stroke patients with and without neglect showed similar impaired reaches to the left side. They concluded that the directional reaching deficits were non-neglect-specific (see also Himmelbach and Karnath, 2003; see Kim et al., 2013 for similar findings and methods but different interpretation of PMN). Noticeably, they report only the group results with high standard errors on their reaching tasks. It is therefore uncertain how the patients performed individually. In other words, it is not clear what percentage of the groups demonstrated reaching deficits to the contralesional side. It is important in this context that not all patients indicate PMN symptoms; therefore, it is uncertain whether a group of patients is representative of PMN. In other words, by diluting the group with patients who do not suffer from PMN, it is not likely to reveal any difference in PMN testing between two groups of right-brain damaged patients that do and do not have neglect (Rorden et al., 2007). This would be evident in a group of neglect patients in which none or only few suffered from PMN. Similarly, Himmelbach and Karnath (2003) criticize various studies (e.g., Husain et al., 2000) that compare reaching deficits in right-brain damaged neglect patients to healthy subjects. To test this point empirically, it would be questionable, for instance, to evaluate a group of patients with neglect in order to explore motor neglect since only a proportion of patients with neglect suffer from motor neglect (Saevarsson, 2013a). Or in Brewer's (1994, p. 119) words: “It is a mistake, in my view, to try to unify the wide variety of phenomena classified as manifestations of “neglect,” by appeal to a single diagnostic or explanatory model of the neglect deficit.” Moreover, Rossit et al. (2009b,a) used mainly the Behavioral Inattention Test (BIT; Wilson et al., 1987) to diagnose neglect in right-hemisphere-injured patients. It is debatable whether to divide participants into neglect and non-neglect subgroups when using the BIT as it does not provide an adequate assessment unless used alongside additional diagnostic resources that are not sensitive to personal and extrapersonal neglect; in addition, the BIT cannot distinguish between the motor and perceptive components of neglect (Plummer et al., 2003). No cut-off scores are given for the BIT and no clear evidence exists for its validity (Cermak and Hausser, 1989). Additionally, therapists sometimes complain that patients perform well on the BIT although their neglect manifests itself clearly in more stressful circumstances in daily life (e.g., Hjaltason and Saevarsson, 2007).

Neuroanatomical evidence against the existence of PMN is infirm and contradictory. Rossit et al. (2009a,b) highlight nodes in the basal ganglia, occipito-parietal cortex, and frontal lobe as being responsible for directional reaching deficits in stroke patients, and claim that these areas are not associated with neglect *per se*, citing the neuroanatomical findings of Karnath et al. (2001, 2004) and Mort et al. (2003). Furthermore, Rossit et al. indicate that damage in the inferior parietal cortex involved in reaching and awareness deficits to the left side was also responsible for directional reaching deficits without neglect. Similarly, Himmelbach and Karnath (2003) hypothesize that the posterior parietal and superior temporal cortex are responsible for directional reaching, and the inferior parietal lobe and superior temporal cortex produce spatial neglect and directional reaching deficits. Many areas of the brain, such as the inferior parietal cortex, temporo-parietal junction (e.g., Mort et al., 2003), superior temporal cortex (Karnath et al., 2004), frontal lobe (Husain and Kennard, 1996; Ghacibeh et al., 2007), and basal ganglia (Karnath et al., 2002; Vossel et al., 2010) are widely believed to be involved in neglect. Therefore, Rossit and Himmelbach et al.'s perspectives differ significantly from other neuroanatomical studies. In other words, by indicating a common neuroanatomical mechanism (e.g. Mattingley et al., 1998; Muggleton et al., 2006), Rossit and others may explain isolated reaching deficits to the left side in neglect. Moreover, Karnath et al. (2001, 2004) and Mort et al. (2003) did not control for directional motor deficits in their studies, therefore making a comparison to the studies of Rossit and Himmelbach and others impossible. Phrased differently, lesion-symptom mapping of two different groups requires symptoms that differ in order to be able to map the area of interest (Rorden et al., 2007). Furthermore, Rossit et al.'s (2009a,b and Himmelbach and Karnath's (2003) sample sizes were only 11, 11, and six neglect patients, respectively, which is likely too small for a meaningful lesion-symptom study. Statistical power is a major concern due to the location distribution of brain lesions (Kimberg et al., 2007). Crucially, there is currently no final agreement on the critical neuroanatomical bases of neglect and PMN due to various methodological assessment issues (see Danckert and Ferber, 2006; Saevarsson, 2013a,b; Saevarsson and Kristjánsson, 2013).

To account for this discrepancy, it is suggested that directional motor deficits observed in right-brain injured patients “without neglect” (who may not suffer from peripersonal visual neglect) indicate PMN that is not coupled with peripersonal visual neglect, or PMN coupled with unspecified visual neglect form. This interpretation is likely since neglect patients commonly indicate double dissociations with respect to visual neglect. For example, Butler et al. (2004) related severity of peripersonal visual neglect to dorsal stream injury and extrapersonal visual neglect to ventral stream damage. Moreover, isolated forms of PMN in right-hemisphere injured patients may be quite common (see Saevarsson and Kristjánsson, 2013 on no neglect improvement following prism adaptation). Indeed, the literature indicates isolated cases of the affliction where only one modality, such as motor or conceptual, is affected (e.g., Laplane and Degos, 1983; Ortigue et al., 2001). Therefore, Himmelbach and Rossit et al. tested right-hemisphere injured patients that may have suffered from an isolated form of PMN and other forms of non-diagnosed neglect. Furthermore, several authors claim that different neuroanatomical mechanisms may explain isolated forms of neglect within the syndrome (e.g., Chechlacz et al., 2012). Coulthard et al. (2006, 2007) argue against the idea that impairments found only in neglect are the sole indication of what the syndrome is. Instead, they assert that neglect is a combination of a group of mental deficits such as impaired spatial memory and directional motor deficits. They explain that PMN can consist of less efficient contralesional reaches and target location on one side, but not to both directions. However, whether and how PMN belongs to the neglect syndrome, should be a central issue when explaining neglect as it affects its assessment and therapy (Saevarsson, 2013b). Indeed, non-sensory factors of movement may be better indicators of poor clinical outcomes than sensory ones (Punt and Riddoch, 2006). PMN and visual feedback are believed to be predictors of successful prism adaptation therapy for neglect (Saevarsson et al., 2009; Striemer and Danckert, 2010a,b; Saevarsson, 2013b; Saevarsson and Kristjánsson, 2013). For instance, Goedert et al. (2014) found bigger improvements on various neglect tests following two weeks of prism adaptation therapy by PMN patients compared to patients suffering from visual neglect without PMN. Similarly, practicing limb movements (Robertson et al., 1992; Pitteri et al., 2013) and increasing contralesional eye movements with prism adaptation intervention improves neglect (Serino et al., 2006). It is also proposed that unspecified frontal and parietal areas play a crucial role in PMN, even if its exact neuroanatomical mechanism is largely not understood. Saevarsson (2013a) reviews 43 studies that apply various assessment approaches and concludes that frontal and parietal structures are most commonly injured in PMN. For instance, Vossel et al. (2010) measured a visual and response bias in neglect with the “turned” manual Landmark task. They found that a visual bias in neglect is caused by frontal, parietal, and occipital injury, while caudate nucleus and putamen were associated with PMN. Mattingley et al. (1998) used a left-right response button task to explore these same components. They show that brain lesions in the inferior parietal lobe—not frontal cortex—explain PMN symptoms and suggest that the inferior parietal lobe operates as a sensorimotor interface. In addition, ignorance of PMN aspects of neglect assessment and the methodological limitations of BIT with respect to neuroanatomical underpinnings call our current understanding of neglect into question (Plummer et al., 2003; Saevarsson, 2013a). Lastly, we call for PMN to be systematically addressed (see Mattingley and Driver, 1997; Saevarsson, 2013a for a discussion and suggestions of PMN assessment) in *every* study on perceptual neglect that requires directional movements because of difficulties in differentiating between the clinical effects of these two subgroups of PMN and visual neglect. One can claim that the critiques of Rossit et al. (2009a) and others are imperfect and that the contralesional directional action components of neglect should remain a part of the standard definition and assessment focus (Saevarsson, 2013a).

## Conflict of interest statement

The authors declare that the research was conducted in the absence of any commercial or financial relationships that could be construed as a potential conflict of interest.
